# Treatment of Aorto-Oesophageal Fistula in a Tertiary German Aortic and Oesophageal Centre A Multidisciplinary Effort

**DOI:** 10.1093/icvts/ivaf236

**Published:** 2025-10-17

**Authors:** Felix Strobl, Jan Stana, Agnes Klara Böhm, Aldin Mehmedovic, Maximilian Pichlmaier, Hubert J Stein, Martin Angele, Nikolaos Tsilimparis

**Affiliations:** Abteilung für Gefäßchirurgie, Klinikum der Universität München, München, 81377, Germany; Klinik für Gefäßchirurgie, Charité Universitätsmedizin Berlin, Berlin, 12203, Germany; Abteilung für Gefäßchirurgie, Klinikum der Universität München, München, 81377, Germany; Klinik für Chirurgie, Charité Universitätsmedizin Berlin, CCM|CVK, Berlin, 13353, Germany; Abteilung für Gefäßchirurgie, Klinikum der Universität München, München, 81377, Germany; Klinik für Herzchirurgie, Klinikum der Universität München, München, 81377, Germany; Klinik für Allgemein-, Viszeral- und Transplantationschirurgie, Klinikum der Universität München, München, 81377, Germany; Klinik für Allgemein-, Viszeral- und Transplantationschirurgie, Klinikum der Universität München, München, 81377, Germany; Abteilung für Gefäßchirurgie, Klinikum der Universität München, München, 81377, Germany

**Keywords:** aorto-oesophageal fistula, TEVAR, endovascular surgery, upper gastrointestinal surgery, cardiothoracic surgery, prosthesis infection

## Abstract

**Objectives:**

Although rare, aorto-oesophageal fistula remains one of the most critical diseases in cardiovascular surgery. The lack of prospective studies or large case series leads to an absence of evidence-based therapeutic concepts.

**Methods:**

We conducted a retrospective analysis of patients treated for aorto-oesophageal fistula between 2014 and 2023. Primary endpoints of analysis were 30-day mortality and median survival; subgroup analysis was performed for aetiology as well as treatment strategy. Additionally, a systematic search was conducted for all studies researching treatment of the disease, including ≥5 patients and published within the last 10 years.

**Results:**

In the collective of 10 patients, 4 manifested as primary fistula, while in 6 patients the fistula occurred secondary to previous thoracic endovascular aortic repair. Median duration to manifestation post-TEVAR was 20.1 months (34.1). Initial treatment consisted of TEVAR or TEVAR-relining in 7 cases, followed by bovine open aortic replacement (*n* = 1) or partial bovine patch repair (*n* = 2) when viable. Treatment of the oesophagus consisted of primary suture (*n* = 1) or oesophagectomy (*n* = 5) with gastric pull-up or colon interposition. Overall 30-day mortality was 40%, and overall median survival was 7.5 months (12.8). Patients receiving surgical treatment of the oesophagus exhibited longer survival than patients who did not (12.8 months [4.7] vs 0.35 months [0.4]). Across the reviewed literature, the strongest effect on survival originates from surgical treatment of the oesophagus. Specific surgical strategies as well as patient characteristics vary widely.

**Conclusions:**

We found TEVAR effective in stabilizing the initial haemorrhage. Short-interval oesophagectomy seems to improve survival and should be considered in most patients. Open aortic replacement with bovine pericardium is a viable option. Interventional treatment options alone do not appear to be sufficient.

## Graphical abstract

**Figure:**
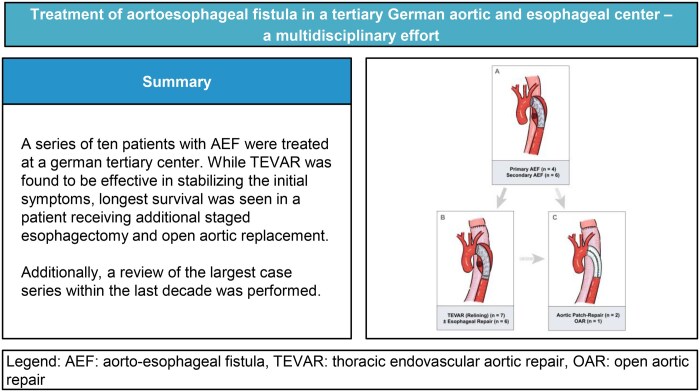

## INTRODUCTION

Aorto-oesophageal fistula (AEF) is an orphan condition with a communicating fistulation between the aorta and oesophagus, with 100% mortality if left untreated.^1–3^ The initially described triad of symptoms—midthoracic pain, sentinel haemorrhage, and exsanguination after an asymptomatic interval—is nowadays of limited applicability. Especially in cases of prior surgical or endovascular aortic repair, fistulation into the aortic lumen is characterized by prosthesis infection more so than active bleeding. Still, mortality under treatment remains strikingly high, with reports ranging from 20% to 90%.^2^^,^^4–14^

Generally, AEF can be divided into a primary and secondary form depending on the underlying pathology. Whereas the primary form is most frequently caused by conditions like thoracic aortic aneurysm, malignant diseases, or foreign body ingestion, the secondary form is defined as a complication following previous thoracic endovascular aortic repair (TEVAR) or open surgery.

While becoming a prominent cause of AEF, TEVAR or TEVAR-relining has also proven the most effective therapeutic measure to control active bleeding. Sole endovascular treatment is, however, not widely considered as sufficient treatment due to the inevitable risk of prosthesis infection. Open surgical replacement of the aorta with biologic material, as is recommended in the European Society of Vascular Surgery’s guidelines for vascular graft infections^15^ is rarely feasible due to the often high fragility of patients upon diagnosis. With prospective studies and subsequent high-level evidence still lacking, optimal treatment modalities are heavily debated. In the following, we present our experiences from a tertiary German aortic and oesophageal surgical centre as well as a systematic overview of the literature of the last 10 years in an attempt to aid in navigating the challenging decision-making process.

## METHODS

A retrospective analysis was performed of all patients treated for AEF at the University Hospital Munich LMU between 2014 and 2023. Patients were identified by searching databases of performed endovascular treatments and oesophageal repairs, as well as lists of diagnoses in the hospital’s information technology system. An approval for the data collection was obtained from the ethics committee (registration number 20-148). Monitoring of ongoing and multiple use of the data is consistent with requirements outlined in the World Medical Assosciation (WMA) Declaration of Taipei. Due to the retrospective nature of the study, written consent was waived. For all identified patients, all available data concerning the hospital stay were collected. Primary outcome measures were defined as 30-day mortality and overall survival. Secondary endpoints included duration of hospital and intensive care unit stay as well as time to oral feeding (when applicable). Follow-up was conducted via our outpatient clinic every 6 months. Alternatively, patients were assessed in subsequent hospital stays, related or unrelated to the underlying disease. No patient was lost to follow-up. Reporting of results was conducted according to STROBE guidelines. Additionally, a systematic search was conducted for all studies researching the treatment of AEF, including ≥5 patients and published within the last 10 years. Databases used, MESH terms, inclusion and exclusion criteria, a PRISM flowchart, as well as a risk-of-bias assessment using Newcastle-Ottawa can be found in the **Supplementary Data**. The review has been registered to PROSPERO under the registration CRD420251070562. Continuous variables are presented as median (IQR). Categorical variables are presented in absolute numbers and percentages.

## RESULTS

Ten patients treated between 2014 and 2023 were identified; baseline characteristics are highlighted in **Table 1**. Median age was 69 years (13.8), with 70% of the studied population being of male sex. The majority of cases occurred secondary to an endovascular, open, or combined (endovascular/open) thoracic aortic repair. Median time of manifestation was 20.1 months (34.1) after the last aortic procedure. Specifics concerning the surgical procedures performed before AEF manifestation can be found in **Table 2**, as well as the detailed case descriptions in the **Supplementary Data**. 40% of cases manifested primarily, mostly after malignant diseases of the oesophagus.

**Table 1.** Baseline Characteristics and Aetiology of AEF

Characteristics	Number of patients
Age	68.5 (13.8)
EuroScore II at diagnosis	11.4 (7.3)
Male sex	7 (70%)
BMI ≥ 30	2 (20%)
Insulin-dependent diabetes	1 (10%)
Hyperlipidaemia	1 (10%)
Active smoker	1 (10%)
Peripheral arterial disease	2 (20%)
Chronic obstructive pulmonary disease	1 (10%)
Reduced left ventricular function	2 (20%)
Previous open aortic surgery	2 (20%)
Previous endovascular aortic surgery	6 (60%)
Primary AEF	4 (40%)
Oesophageal carcinoma	2 (20%)
AEG tumour	1 (10%)
Ruptured thoraco-abdominal aneurysm	1 (10%)
Secondary AEF	6 (60%)
TEVAR	3 (30%)
Bentall + TEVAR	2 (20%)
Branched TEVAR	1 (10%)

Age and EuroScore II are presented as median (IQR).

Abbreviations: AEF, aorto-oesophageal fistula; AEG, adenocarcinoma of the oesophagogastral junction; BMI, body mass index; TEVAR, thoracic endovascular aortic repair.

**Table 2.** Clinical Presentation

Patient No.	Aetiological category	Underlying disease	Previous treatment	Time since last operation (months)	Initial presentation	Height of fistula (cm off TR)	Microbiology	Antibiotics	Aortic procedure	Prosthesis used	Oesophageal procedure	EndoVAC	Survival after diagnosis (months)	Cause of death
1	Secondary	Inflammatory TAA	Branched TEVAR	34.7	Inflammation	30	VRE, C. albicans	n/a	Pericardium patch	/	Oesophagectomy with gastric pull-up	Yes	0.7	Sepsis
2	Primary	Oesophageal-Ca	/		Haematemesis	29	/	Pip/Taz; Metro/Fluco	TEVAR	ZDEG 26-136 ZT	Oesophageal stenting (20 mm PTFE)	No	0.2	Sepsis
3	Primary	Oesophageal-Ca	Radio-chemotherapy		Haematemesis	26	/	Pip/Taz; Vanco/Caspo	TEVAR	ZTEG-2P-34-152-PF	/	No	0.5	Sepsis
4	Secondary	Ruptured TAAA	TEVAR	1.2	Inflammation	38	*Candida kefyr*, *Mukaplasma salwarii*	Mero/Vanco/Micafungin; Doxy/Fluco	TEVAR-relining, open aortic replacement with pericardium	ZEG 30-141	Oesophagectomy with gastric pull-up, colon interposition	Yes	38.2	/
5	Secondary	Type A dissection, post-dissection TAA	Bentall operation, TEVAR	1.8	Haematemesis, shock	35	VRE	Mero/Caspo, Tige/Line/Dapto	1fTEVAR-relining	ZDEG-PT-38-154 (surgeon modified fenestration for left CCA)	Oesophagectomy with esophagostoma	No	13.9	Malignant disease
6	Primary	AEG	Esophagectomy with gastric pull-up		Haematemesis, shock	26	/	Mero/Vanco	TEVAR	ZDEG P 32 202 PF	Sengstaken-Blakemore	Yes	6.1	Recurrent bleeding
7	Primary	Ruptured TAAA	/		Inflammation	42	*Escherichia coli*, *Lactobacillus rhamnosus*, *and Candida albicans*	Mero/Vanco/Fluco	TEVAR	ZDEG P 30 147 ZDEG P 32 202 surgeon modified fEVAR ZDEG 32 142 PF aortal pericardium patch	Primary suture, omental patch	Yes	14.4	/
8	Secondary	Ruptured TAA	TEVAR + debranching	37.6	Thoracic pressure, dysphagia	25	/	Pip/Taz; Mero/Vanco/Caspo	TEVAR-relining, haematoma clearance	ZTA-PT-42-38-255 ZTA-P-40-167	Transmediastinal oesophagectomy with gastric pull-up	No	11.7	/
9	Secondary	Ruptured TAA (after Ib endoleak)	TEVAR + TEVAR-relining	5.6	Haematemesis, shock	/	/	/	/	/	/	No	0	Acute bleeding
10	Secondary	Type A dissection, post-dissection TAA	frozen elephant trunk, fEVAR	70.1	Inflammation	30	*Streptococcus anginosus*	Pip/Taz; Mero/Vanco	/	/	Oesophagectomy with gastric pull-up	No	8.9	/

Abbreviations: AEG, adenocarcinoma of the oesophagogastral junction; Ca, carcinoma; Caspo, caspofungin; CCA, common carotid artery; Doxy, doxycycline; EndoVAC, endoluminal vacuum-assisted closure; fEVAR, fenestrated endovascular aortic repair; Fluco, fluconazole; Line, linezolid; Mero, meropenem; Metro, metronidazole; Pip/Taz, piperacillin/tazobactam; PTFE, polytetrafluoroethylene; TAA, thoracic aortic aneurysm; TAAA, thoracoabdominal aortic aneurysm; TEVAR, thoracic endovascular aortic repair; Tige, tigecycline; TR, teeth row; Vanco, vancomycin; VRE, vancomycin-resistant enterococcus.

Most patients initially presented with haematemesis (60%), half in haemorrhagic shock. Three patients presented with nonspecific signs of inflammation, while one patient’s primary symptoms were thoracic pressure and dyspnoea.

**Figure 1.**
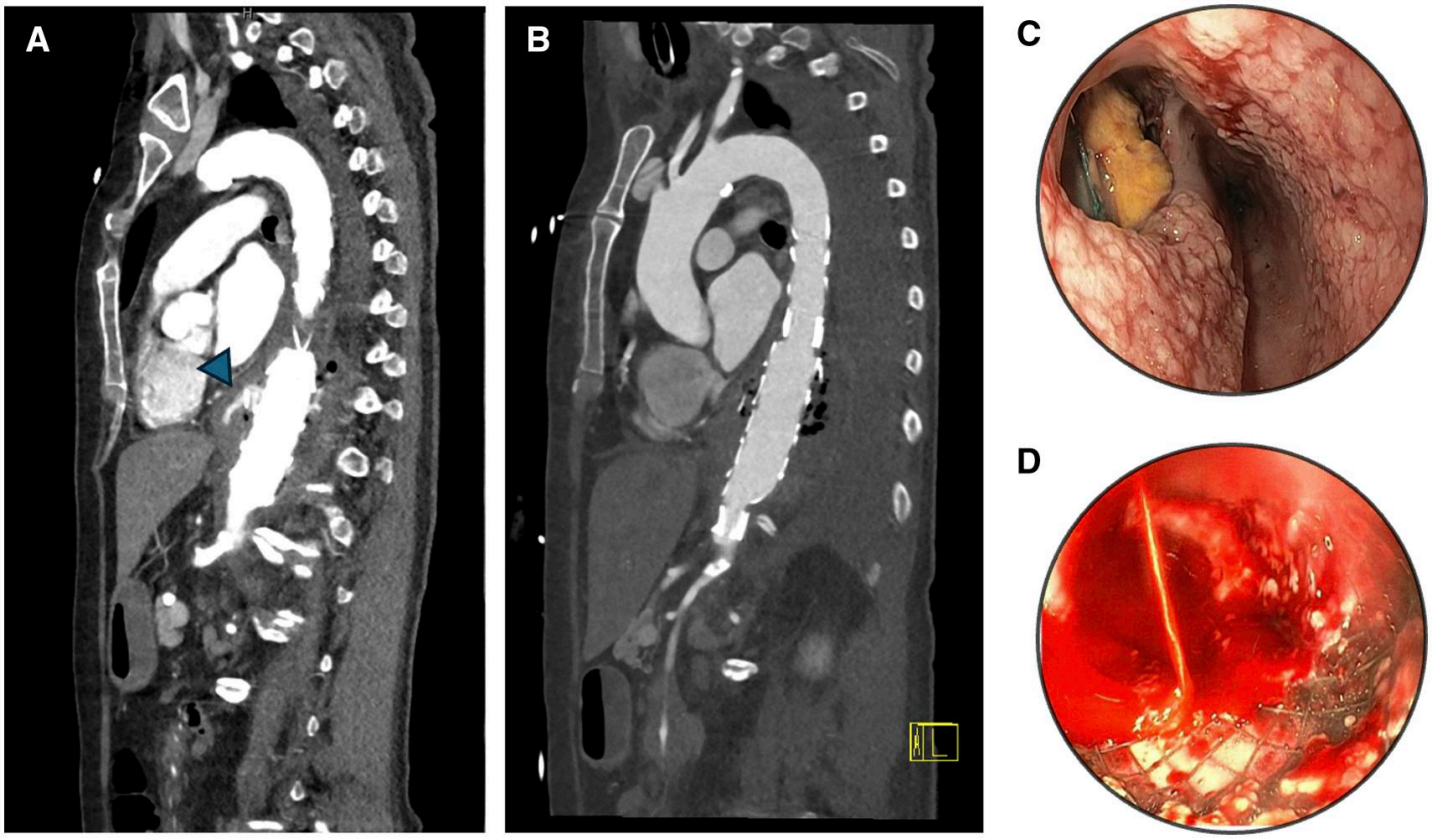
CT Scans and Endoscopy Images of AEF. (A) AEF in patient #4 before and (B) after TEVAR-relining. The arrow shows active bleeding. (C) Esophagoscopy of patient #4 after TEVAR-relining. The aortic stent is clearly visible in the mediastinum. (D) Endoscopic image of the oesophagus of patient #2 with oesophageal stent *in situ*. Abbreviations: AEF, aorto-oesophageal fistula; TEVAR, thoracic endovascular aortic repair

Contrast-enhanced CT of the aorta was performed in all patients. Air trapped in the aneurysm as a sign of prosthesis infection was considered a strong indicator; arterial bleeding into the oesophagus was a secure diagnostic criterion. If the patient’s cardiopulmonary situation allowed, gastroscopy was performed to confirm diagnosis. An exemplary CT scan, as well as endoscopic images of an actively bleeding and a non-bleeding fistula, can be found in **Figure 1**. When gastroscopically visible, the height of the perforation was documented. It was seen at a median of 30 (9) cm from the teeth row. In some cases, positron emission tomography was added to support diagnosis of prosthetic infection. In 2 cases, gastroscopy was also attempted in a therapeutic effort, applying an oesophageal stent or a Sengstaken-Blakemore tube. Both patients with tumour-related AEF could not be sufficiently stabilized by this procedure and received TEVAR shortly after. Out of all patients, 70% received TEVAR or TEVAR-relining as initial therapy. Endoprostheses were soaked in rifampicin before implantation. Of patients not receiving TEVAR, 1 patient (#9) passed before reaching the operating room (OR), 2 patients were not showing active signs of bleeding and were thus scheduled for short-interval open surgery without bridging TEVAR.

**Figure 2.**
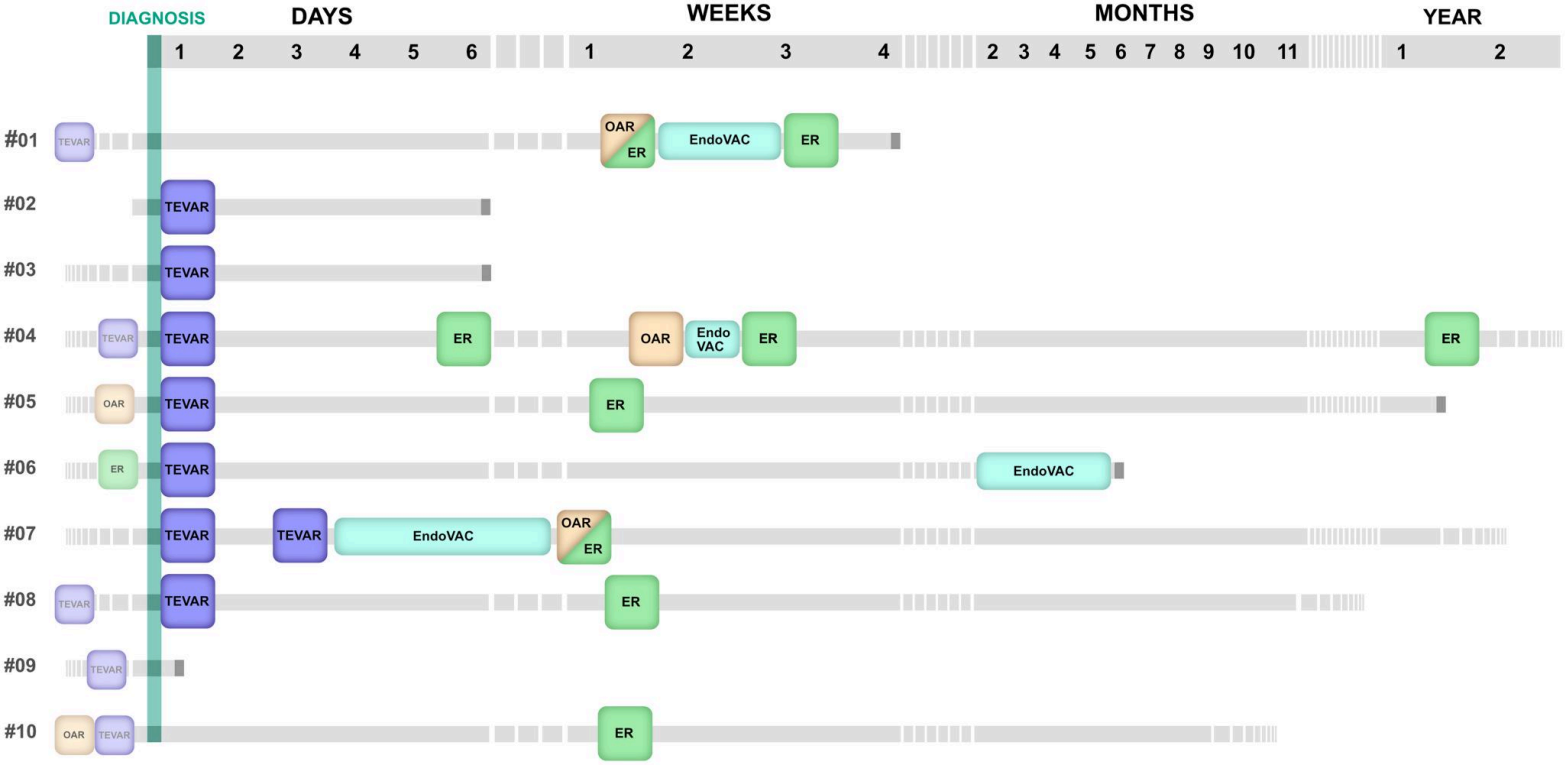
Graphical Timeline Illustrating Cases. Termination of Line Indicates Death

Treatment, as well as preoperative state and survival, is visualized in the graphical timeline (**Figure 2**). Out of all patients treated, 6 received oesophagectomy (*n* = 5) or oesophageal suture (*n* = 1) with omental patch in the short-term course of hospitalization. Reconstruction was mostly performed immediately after a successful gastric pull-up (*n* = 4). One patient received permanent discontinuity resection after cervical oesophagostomy, another had to be revised due to an insufficiency of the gastroesophagostomy and received a discontinuity resection followed by a colon interposition after readmission several months later. Three out of the patients receiving surgical treatment of the oesophagus were also treated by endoluminal vacuum-assisted closure (EndoVAC) therapy, usually preoperatively to reduce infection and precondition tissue. In 1 patient, EndoVAC therapy alone was attempted because of a lack of surgical options; however, it did not contain prosthesis infection sufficiently.

In patients who were evaluated as stable enough, as well as lacking terminal disease limiting life expectancy, open aortic repair with pericardial patch leaving the stent in situ (*n* = 2) or stent graft removal and descending aortic replacement with tubularized pericardium were performed (*n* = 1).

Median overall patient survival is 7.5 months (12.8) at the time of this study, with 4 patients alive and in regular follow-up care 8.9 to 38.2 months following repair of AEF. The leading cause of death was sepsis (3/6 cases), followed by acute or chronically recurrent bleeding.

Patients with secondary AEF showed more complicated courses of disease, with longer hospital (17 days [12] vs 42 days [30]) and intensive care unit (ICU) stays (8 days [5] vs 27 days [22]). 30-day mortality rates were 66% compared to 50% in patients with primary AEF.

In terms of treatment for the oesophagus, patients who received surgical intervention showed markedly longer hospital stays (48 days [22] vs 11 days [12]), as well as lower 30-day mortality (50 vs 75%) and longer overall survival (12.8 months [4.7] vs 0.35 months [0.4]) than patients receiving only stenting or no treatment of the oesophagus.

Longest survival is documented for patients #4 and #7, both receiving emergency TEVAR followed by (1) oesophagectomy (plus intermittent endo-sponge therapy) and (2) staged or concomitant open aortic repair. While the former patient received stent removal and total replacement of the descending aorta, the latter only received a partial replacement and is currently stable under antibiotic suppression therapy. The shortest survival is described in patients either passing before reaching the OR for emergency TEVAR (patient #9) or patients with a shift towards a palliative care goal after initial stabilization due to underlying late-stage malignancy (patients #2 and #3).

Broad antimicrobial treatment was commenced in all patients; individual protocols are, however, not always documented sufficiently. Microbial analyses were performed on blood cultures, as well as swabs in case of subsequent open surgery (**Table 2**).

The systematic search of relevant original research articles published in the last 10 years focusing on surgical treatment of AEF resulted in 10 case series and a total of 111 patients; key results are summarized in **Table 3**. All database and questionnaire studies were excluded. Patient populations ranged from 6 to 16 patients, exhibiting high inhomogeneity concerning aetiology of the fistulas within the respective studies. Two studies focused strictly on secondary AEF; of the remaining 8 studies, 4 included tumour-related AEF as well. Across studies, early mortality ranged from 20% to 60%, with a 100% mortality in patients receiving no surgical treatment. Overall mortality ranged from 20% to 90%; however, only 4 of the studies specified follow-up times beyond visualization in Kaplan-Meier plots, ranging from 4.2 to 39 months. Most groups saw the best results in patients receiving radical surgical treatment of the oesophagus, as well as opting for a staged hybrid repair of the aorta using TEVAR first and open repair after stabilization. Statistical analyses between subgroups are rare due to small sample sizes.

**Table 3.** Overview of All Single-Centre Studies with ≥5 Patients with AEF Conducted in the Last 10 Years

Study	Time frame	*N*	Aetiology	Secondary	Treatment strategies	*N*	Early mortality	Overall mortality	Follow-up (months)	Comment
Strobl 2025	2014-2023	10	Secondary (6): (b)TEVAR (4), OAR + TEVAR (2) Oesophageal/gastric cancer (3) Aortic rupture (1)	60%	TEVAR + ER OAR + ER (±TEVAR) ER TEVAR NST **Overall**	2 3 1 3 1 **10**	0% 50% 0% 66% 100% **40%**	50% 50% 0% 100% 100% **60%**	12.8 17.8 8.9 2.3 / **7.5**	
Jeon 2022	2007-2018	10	Secondary (6): TEVAR (3), OAR (3) Aneurysm (2) Aortic rupture (1) Oesophageal rupture (1)	60%	Aortic homograft (±bTEVAR) + ER Prosthetic aortic graft (±bTEVAR) + ER Extra-anatomical bypass + ER **Overall**	5 4 1 **10**	0% 50% 0% **20%**	0% 50% 0% **20%**	n/a	ER was performed as primary repair or oesophagectomy. All patients who underwent oesophagectomy (*n* = 3) survived.
Chen 2021	2005-2018	8	Secondary (1): OAR (1) Oesophageal/gastric cancer (6) Oesophageal tuberculosis (1)	12.5%	OAR + ER TEVAR TEVAR + EMB EMB NST **Overall**	1 2 2 1 2 **8**	0% 50% 0% 100% 100% **50%**	0% 50% 100% 100% 100% **75%**	n/a	Embolization was performed using N-butyl cyanoacrylate. Patients receiving TEVAR + EMB died of unrelated causes.
Omran 2021	2011-2019	10	Secondary (2): OAR (2) Barrett’s oesophagus (1) Oesophageal/gastric/breast cancer (7)	20%	TEVAR ± ER/stenting TEVAR + OAR NST **Overall**	8 1 1 **10**	50% 0% 100% **60%**	87.5% 100% 100% **90%**	3.1 6 **4.2**	Evaluation of AEF and ABF. Three of the cancer patients developed AEF after EndoVAC therapy. One patient was deemed inoperable.
Sugiyama 2020	2011-2016	6	Secondary (6): TEVAR (4), OAR (2)	100%	TEVAR OAR + ER TEVAR + ER ± extra-anatomical bypass **Overall**	2 2 2 **6**	0% 0% 100% **33%**	50% 50% 100% **66%**	n/a	
Yamazato 2018	1999-2017	18	Aneurysm (12) Aortic dissection (6) Of these secondary (12): TEVAR (7), OAR/arch replacement (5)	66.6%	Aortic treatment TEVAR ± OAR (±omentopexy) OAR (±omentopexy) Oesophageal treatment ER (simultaneous or staged) No ER **Overall**	6 12 16 1 **18**	**22%**	**42%**	**22.5**	The study did not conduct comparisons between treatment groups (high inhomogeneity). Oesophageal reconstruction with jejunum (8) or ileocecum (1) ± supercharged anastomosis. 13 patients receiving in situ reconstruction of the aorta also received omentopexy.
Kawamoto 2015	2005-2013	10	Secondary (6): TEVAR (2), OAR (2), TEVAR + OAR (2) Aneurysm (2) PAU (2)	60%	TEVAR + ER + OAR TEVAR + ER OAR + ER **Overall**	8 1 1 **10**	25% 100% 0% **30%**	50% 100% 0% **50%**	n/a	Oesophageal reconstruction in 6 cases with colon (3), jejunum (2) or gastric pull-up (1) ± supercharged anastomosis.
Luehr 2015	2002-2013	8	Secondary (8): TEVAR (8)	100%	OAR + ER ER NST **Overall**	6 1 1 **8**	16.6% 100% 100% **37.5%**	n/a	n/a	
Mosquera 2014	1998-2013	8	Secondary (2): TEVAR (1), OAR (1) Aortic rupture (1) Aneurysm (2) Oesophageal cancer (3)	25%	TEVAR NST **Overall**	3 5 **8**	**75%**	**87.5%**	n/a	Evaluation of AEF and ABF. Overall better survival in secondary manifestations.
Kahlberg 2014	2006-2013	7 [11]	Secondary (4) PAU (1) Aneurysm (1) Foreign body ingestion (1)	57.1%	TEVAR + ER with muscle flap interposition [NST] **Overall**	7 [4] **11**	0% [100%] **36.4%**	14.2% [100%] **45.4%**	39	Numbers in square brackets show results including patients who died upon arrival (excluded from the study)
Okita 2014	1999-2013	16	Secondary (4): TEVAR (4) Aneurysm (4) Dissection (1) Oesophageal cancer (5) Foreign body ingestion (1)	26.6%	TEVAR OAR + ER TEVAR + OAR + ER NST **Overall**	5 4 4 1 **15**	40% 0% 0% 100% **20%**	100% 50% 50% 100% **73.3%**	2.25 10.65 2.85 6.7 **6.7**	

Early mortality consists of 30-day mortality or in-hospital mortality (whichever is specified). Follow-up presented as mean.

Abbreviations: ABF, aorto-bronchial fistula; AEF, aorto-oesophageal fistula; bTEVAR, branched thoracic endovascular aortic repair; EMB, embolization of fistula; EndoVAC, endoluminal vacuum-assisted closure; ER, oesophagectomy/oesophageal repair; n/a, not available; NST, no surgical treatment; OAR, open aortic repair; PAU, penetrating aortic ulcer; TEVAR, thoracic endovascular aortic repair.

## DISCUSSION

AEF remains a condition with substantial lethality; our cohort showed a 30-day mortality rate of 40%. We found TEVAR most effective in stabilizing active bleeding in patients with tumour-related as well as non-tumour-related AEF. Patients receiving surgical treatment of the oesophagus presented with longer ICU and hospital stays; however, they also showed longer median survival times. In our cohort, open aortic replacement after TEVAR was only performed in 1 patient using tubularized bovine pericardium, representing the only case reaching what could be considered long-term survival (>3 years). TEVAR as definitive treatment of the aorta was chosen in several patients due to underlying conditions preventing further surgical intervention, especially patients with late-stage malignancies.

AEF can be challenging to diagnose in an adequate timeframe due to its oftentimes nonspecific presentation. In secondary AEF cases, clinical presentation was more likely to consist of symptoms of prosthesis infection instead of active bleeding. Clinicians should be aware of this phenomenon and initiate diagnostics and transfer to a referral centre for both aortic and oesophageal surgery immediately upon suspicion of AEF. Lesions are best visible by gastroscopy and typically occur below the tracheobronchial constriction, which seems characteristic of the disease, due to the anatomic proximity of the oesophagus and aorta.

Following ruptures at the level of the mid-thoracic aorta, special awareness needs to be present regarding the risk of AEF due to local compression of the oesophagus. Pseudoaneurysms of the thoracic aorta in this area should also raise the suspicion of a primary oesophageal pathology with secondary erosion of the aorta.

The single-centre studies included in the systematic review tended to be descriptive in nature with highly heterogeneous patient populations (**Table 3**).^5–14^ General consensus tends to favour surgical intervention overall, as early mortality of patients not receiving surgical treatment is 100% across studies. Usually, TEVAR is favoured for controlling active bleeding; however, it is rarely employed as a definitive surgical treatment.^7^^,^^8^^,^^12^^,^^14^ It is commonly followed by short-interval oesophagectomy or open oesophageal repair,^5–11^^,^^13^^,^^14^ as well as removal of the stent with consecutive open aortic replacement to mitigate stent infection.^5^^,^^14^ In secondary AEF cases without active bleeding, bridging-TEVAR was foregone by multiple groups with no obvious effect on short-term mortality.^5^^,^^8^^,^^9^^,^^11^ Interventional treatment options, such as embolization of the fistula, are rarely employed and do not show promising results.^7^

Most groups performed oesophagectomy with primary or interval reconstruction using gastric pull-up or colon-/small bowel interposition. In some cases, reconstruction is performed using *super-charged* anastomoses—a pedicled vascular anastomosis is added to lower the risk of anastomotic insufficiency.^4^ In certain instances, oesophageal repair was also performed without oesophagectomy using omental wrapping or muscle flap interposition; however, there is no clear evidence for either augmentation.^9^^,^^13^ No data are available on the timing of oesophagectomy after diagnosis; however, some colleagues recommend performing surgery as soon as possible after stabilization of the patient to avoid stent infection.^1^^,^^2^^,^^4^^,^^16^

Jeong et al. report very low mortality rates of 20% in a collective of 10 patients treated by bridging-TEVAR and subsequent oesophagectomy, as well as open aortic replacement with removal of the aortic stent and either homograft or synthetic prosthesis. While these results seem very promising, we do suspect a certain selection bias towards a healthier than average patient collective for all of them to be considered for open aortic repair. Of note, all of the patients receiving homografts survived until the end of follow-up, suggesting longer overall survival.^5^ In the context of aortic graft infections in general, replacement of the aorta with tubularized bovine pericardium, as employed by our group, has recently been shown in a multicentric study to yield similarly low re-infection rates as do conventional replacements with aortic homografts.^17^ Other groups have also reported favourable results employing extra-anatomical bypassing of the aorta, thus avoiding placing synthetic material in the infected site altogether.^8^^,^^14^

Kahlberg et al. also reported on high survival rates (86%) in patients only receiving TEVAR and oesophageal suture with intercostal muscle flap interposition after a median of 39 months.^13^

Consistent with our findings, Okita et al. also reported the best outcomes for patients receiving oesophagectomy as well as aortic replacement in their collective of 16 patients; however, they mostly used Dacron or polytetrafluoroethylene prosthesis in situ or extra-anatomically. They recommend performing surgery within 1 week of TEVAR and subsequent lifelong antibiotic treatment of these patients.^14^

The largest multicentric databank analysis to date by Czerny et al., including 36 cases, favours surgical treatment in general over conservative treatment (46% vs 100% mortality) without analysing sub-groups.^2^ Two studies collected results across multiple centres in Japan via questionnaires with a total collective of 47 and 39 patients, respectively.^18^^,^^19^ Akashi et al. found oesophagectomy, omental wrapping, and open aortic repair as independent predictors of higher survival after 6 and 18 months.^18^ Watanabe et al. found significantly longer survival in patients with secondary AEF when receiving oesophagectomy.^19^

Several reviews collecting case reports and series also deal with the question of optimal treatment algorithm; however, they should be evaluated carefully due to being affected by publication bias and incomplete reporting.

Jonker et al. analysed 43 cases of AEF published online and found that patients receiving oesophagectomy within 30 days after TEVAR presented significantly lower mortality.^20^ Takeno et al. analysed 172 cases and found significantly longer survival in patients receiving oesophagectomy, but also significantly favoured open aortic replacement after TEVAR over TEVAR alone.^1^ Canaud et al. analysed 72 case reports and found systemic antibiotic treatment >4 weeks to be the only statistically significant factor influencing survival.^4^ In our opinion, broad antimicrobial therapy—ideally adapted to swab results, alternatively calculated for oesophageal microbiome—should be commenced early and continued long-term. As a standard regimen, we would suggest initial calculated therapy using a combination of meropenem, vancomycin, and caspofungin. De-escalation can follow upon discharge and should be continued long term.

Our proposed therapeutic algorithm involves a multidisciplinary approach. Treatment should be centralized in specialized hospitals, and every case should be regarded as a highly patient-tailored approach. Patients and relatives should be made aware of the dire prognosis even under maximally aggressive therapy.

Several limitations of this study need to be addressed. Due to the nature of retrospective analysis, the absence of a control group, small sample size, and high heterogeneity in the patient collective, especially including patients with tumour-related AEF, the conclusions drawn must be treated with caution. Due to generally short follow-up times (only 4 patients >1 year), no conclusions concerning long-term survival can be drawn. Concerning the favourable results in patients receiving more aggressive surgical treatment, due to high perioperative risk, a selection bias towards a healthier than average patient collective is possible. Concerning the literature review, although it has generally been carried out with adherence to the PRISMA guidelines, it does not fulfil all requirements for a systematic review. Additionally, due to the nature of available articles, it consists exclusively of small observational studies without control groups, leading to a substantial risk of bias. Due to the lack of available data, individual treatment regimens had to be categorized to allow for any comparisons between studies. Exact follow-up times were also rarely mentioned in the studies.

## CONCLUSION

Emergency TEVAR is an effective strategy to initially stabilize patients who present with haemorrhage. Our results, in line with currently available literature, seem to favour more invasive therapy. Especially, surgical treatment of the oesophagus should always be considered if feasible by the patient comorbidities. If stent removal is within an acceptable perioperative risk, it is technically feasible to use customized grafts out of bovine pericardium patches for open aortic repair.

## Supplementary Material

ivaf236_Supplementary_Data

## Data Availability

Additional data to support the findings are included in the supplementary data file.
