# Supplementary material for: Treatment of Aorto-Oesophageal Fistula in a Tertiary German Aortic and Oesophageal Centre A Multidisciplinary Effort
Source: Interdiscip Cardiovasc Thorac Surg. 2025 Oct 17;40(11):ivaf236. doi: 10.1093/icvts/ivaf236 (PMC12622961; doi:10.1093/icvts/ivaf236)
Supplement: ivaf236_Supplementary_Data [file ivaf236_supplementary_data.zip › Supplementary Data Revision 3.docx]

Supplementary Tables and Figures

**Supplementary Figure 1 – Kaplan Meier Analysis of survival based on etiology, surgical treatment of the esophageal lesion and initial vascular treatment.** Statistical analyses performed using log-Rank Test.

|  |  |  |  |  |  |
| --- | --- | --- | --- | --- | --- |
| **Outcome** | **total** | **Primary AEF**  **(n = 4)** | **Secondary AEF**  **(n = 6)** | **Esophagectomy / esophageal repair**  **(n = 6)** | **Esophageal stenting / no treatment**  **(n = 4)** |
| Hospital stay (d) | 26 (35) | 17 (12) | 42 (30) | 48 (22) | 11 (12) |
| ICU stay  (d) | 12 (23.5) | 8 (5) | 27 (22) | 27 (18) | 6 (4) |
| Time to oral feeding (d) | 23 (17)  (n=5) | 19 (5)  (n=2) | 31 (322)  (n=3) | 27 (167)  (n=4) | 14  (n=1) |
| 30-day mortality | 40% | 50% | 66% | 50% | 75% |
| Median survival  (month) | 7.5 (12.8) | 3.3 (7.8) | 10.6 (10.3) | 12.8 (4.7) | 0.35 (0.4) |

**Supplementary Table 1 – Subgroup analyses.** Reported as Median (IQR), or percentages of total.

Case descriptions

The first patient – a 75-year-old male – presented with elevated inflammatory parameters and persistent stomach aches 34 months after endovascular thoracic aorta reconstruction (Carotid-subclavian bypass plus TEVAR). A CT showed severe mediastinitis, an initial conservative therapy with antibiotics and antimycotics ensued. After stabilization and evaluation of operability, transhiatal esophagectomy with gastric pull-up and primary repair of the aorta with a pericardium patch were performed on day ten after admission. Intraoperative swabs of the mediastinum showed ﻿Vancomycin-resistant Enterococcus faecium and Candida albicans. Eight days post-operation, an insufficiency of the esophago-gastrostomy was detected endoscopically. Neither endoluminal vacuum therapy nor operative revision of the anastomosis with introduction of a rinse-suction drainage were sufficient in controlling the inflammation. The patient died due to fulminant sepsis 32 days after diagnosis of AEF.

The second patient – a 57-year-old male – presented with hematemesis 38 months after initial diagnosis of inoperable esophageal cancer treated by radio-chemotherapy. Massive uncontrollable hemorrhage was seen endoscopically at the distal end of the previously introduced esophageal stent. A CAT-scan confirmed the diagnosis of AEF, the patient received TEVAR (Cook ZDEG 26-136 ZT) for hemostasis after needing reanimation and mass transfusion because of hypovolaemic shock. Due to oncologic inoperability, a covered esophageal stent was introduced in an effort to seal the perforation of the esophagus. The patient’s relatives decided against any further invasive treatment, leading to the patient’s death 6 days after diagnosis.

The third patient – a 78-year-old female with a history of esophageal cancer – presented with hematemesis 6 months after initial cancer diagnosis, and previously treated by neoadjuvant radio chemotherapy. The patient was treated by TEVAR (ZTEG-2P-34-152-PF), achieving hemostasis but occluding the left subclavian as well as vertebral artery. Within days after the operation, the patient developed elevated inflammatory parameters, most likely due to aspiration pneumonia. Further treatment was declined by the patient, ending in the patient’s death by sepsis within 6 days of diagnosis.

The fourth patient – a 71-year-old female – presented in our emergency department with elevated inflammatory parameters 38 days after receiving TEVAR (ZTA P 28-155) for ruptured thoracoabdominal aortic aneurysm. After initial emergency TEVAR-relining with a larger sized prothesis (ZEG 30-141), thoracoabdominal esophagectomy with gastric pull-up was performed 6 days later. Due to persistent septic constellation, surgical treatment of the esophagus was followed by open stent removal and aortic replacement using a rolled up bovine pericardium patch (tube) 13 days after diagnosis. Because of insufficiency of the esophago-gastric anastomosis (indicated by massive leucocytosis), a discontinuity operation with cervical esophagostomy had to be performed 10 days after the initial operation. 16 months after esophagostomy, GI-continuity was reconstructed using colon interposition. After achieving successful oral feeding there was a need to place a percutaneous endoscopic gastrostomy (PEG) due to persisting dysphagia six months after the second reconstruction. The patient is alive at the time of last follow-up after 38.2 months.

Patient no. five – a 54-year-old male – presented with hematemesis and cardiocirculatory shock 55 days after receiving TEVAR additionally to a Bentall operation for Stanford A dissection 9 months prior. Because of a persisting endoleak type 1A and 1B, the patient received a surgeon modified fenestrated TEVAR-relining (ZDEG-PT-38-154, fenestration for left common carotid artery) as initial treatment. As treatment for the esophagus, two covered stents were placed in the area of perforation. Because of persisting fistulation, discontinuity resection with esophagostomy and PEG tube was chosen as definitive treatment. Restoration of continuity was initially omitted, as a PET-Scan showed persistent inflammation in the aortal prothesis. Open aortic replacement was not considered feasible for the patient due to extensive previous operations and reduced general condition. The patient suffered recurring septic episodes refractory to antibiotic treatment. For lack of curative options, the patient was transferred to palliative care and finally passed 13.9 months after initial diagnosis.

The 64-year-old male sixth patient was admitted with hematemesis and subsequent cardiopulmonary instability about 1 month after esophagectomy with gastric pull-up as treatment for an AEG tumor. Bleeding was controlled initially using a Sengstaken-Blakemore tube and an emergency TEVAR (ZDEG-P-32-202-PF), achieving hemodynamic stability. Perforation was located at the gastro-esophageal anastomosis. Due to limited further surgical option for the esophagus, Endo-VAC therapy was subsequently employed to treat prothesis infection. After initial recovery and discharging the patient with oral food intake, readmission became necessary within 3 months because of recurrent bleeding. Due to the advanced state of the oncological disease and limited treatment options, palliative care was provided, and the patient passed away 6.1 months after diagnosis.

The 69-year-old male seventh patient was admitted with a contained ruptured aortic aneurysm in the descending aorta. Initially, the patient presented in a peripheral hospital with epigastric pain for two days and massively elevated inflammatory parameters. The patient was subsequently transferred and immediately provided with TEVAR (2x ZDEG-P-30-147). In a gastric scope AEF was diagnosed at 42 cm off row of teeth, with consolidated hematoma and no active bleeding. Within a two-day interval, the patient received treatment for the remaining aneurysm of the abdominal aorta with a 4-vessel physician modified fenestrated aortic prothesis (physician modified ZDEG-P-32-202). Within an interval of 5 days, the esophagus was repaired using direct suturing, an omental patch and local drainage and flushing. The defect of the aorta was covered using a bovine pericardium patch, however leaving the stents in place. Endo-VAC treatment was performed additionally. After 23 days, the patient recovered to allow for oral feeding and was discharged 42 days after admission with long term antibiotic treatment. The patient is currently alive and in regular follow-up after 14.4 months.

The 68-year-old female eighth patient presented with thoracic pain and dysphagia 37 months after TEVAR with prior carotid-carotid and carotid-subclavian bypass for treatment of aortic arch aneurysm in an outside institution. An initial CT upon admission showed a large mediastinal hematoma compressing the esophagus probably as a result of aortic rupture, whereas a subsequent gastroscopy disproved the existence of AEF at this point. No obvious endoleak was visible at this point. After 4 days, a follow-up gastroscopy revealed progressive indentation of the esophagus by an increasing hematoma and thinning of the wall, without CT-morphologically visible aortic perforation. The patient received TEVAR relining (using ZTA-PT-42-38-255 and ZTA-P-40-167) and subsequent additional open evacuation of the hematoma. After initially stabilizing the patient’s condition, another follow-up gastroscopy was performed 7 days after the operation due to elevated inflammatory parameters and showed esophageal perforation at 25 cm off teeth row. Esophagectomy and reconstruction by gastric pull-up was performed emergently. The patient recovered, reached full oral feeding after 31 days and is alive at the time of last follow-up after 11.7 months.

The 77-year-old male ninth patient initially received TEVAR for aortic aneurysm and TEVAR relining 23 months later due to endoleak before presenting with pronounced dysphagia 42 days after the second intervention. Gastroscopy showed esophageal stenosis and thinning of the wall at 42 cm off teeth row, caused by indentation through the aneurysm. Due to underlying cardiopulmonary condition, the patient was deemed unsuitable for esophagectomy or open repair. Dilation of the stenosis was purposely omitted so as not to provoke perforation. A PEG tube was placed for enteral nutrition and the patient was discharged. The patient was readmitted 4 months later with hematemesis and hypovolemic shock. After gastroscopy confirmed massive arterial bleeding, decision towards palliative care was made due to lack of therapeutic options.

The 59-year-old male tenth patient presented with elevated inflammatory parameters 30 months after fenestrated EVAR (fEVAR) for post dissection thoraco-abdominal aneurysm and 14 years after receiving Bentall-operation for Stanford A dissection. Intravenous broadband antibiotic therapy was started. After 19 days, PET-CT showed signs of prothesis infection and subsequent gastroscopy showed AEF 30 cm off teeth row. Nine days later, esophagectomy with gastric pull-up was performed. The patient reached full oral feeding 13 days post operation and was discharged with lifelong oral antibiotic treatment consisting of amoxicillin and fluconazole. The patient was readmitted for hepatic bleeding due to strict anticoagulation needed for his artificial valve. The patient is alive at last check-up after 8.9 months, however suffering from lower extremity weakness due to late onset spinal ischemia.

Systematic Search

Cue

(Aortoesophageal*[tiab] OR Aortooesophageal*[tiab] OR Aorto-esophageal*[tiab] OR Aorto-oesophageal*[tiab] OR "aortic" OR ("aorto" AND ("esophageal" OR "oesophageal"))) AND (fistul*[tiab]) AND ("endovascular" OR "TEVAR" OR "Thoracic endovascular aortic repair")

Article types

Randomized controlled trial

Comparative study

Observational study

Case series

Timeframe

≥ 01/01/2014

Search conducted

27/04/2024

Excluded due to publication date (exceeding last 10 years)

n = 571

Records identified from MEDLINE library through PubMed

n = 1266

**Identification**

Studies excluded due to non-elligible type of publication (e.g. review, lecture)

n = 274

Records screened

n = 695

**Screening**

Reports excluded due to

- Therapeutic strategies of AEF not main focus
- No. of patients < 5
- Questionnaire studies
- Language except English
- No abstract/full text available

n = 411

Reports assessed for eligibility

n = 421

Studies included in final qualitative synthesis

n = 10

**Included**


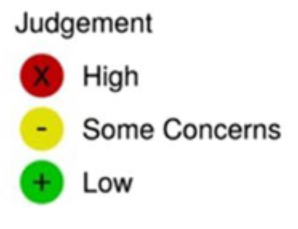

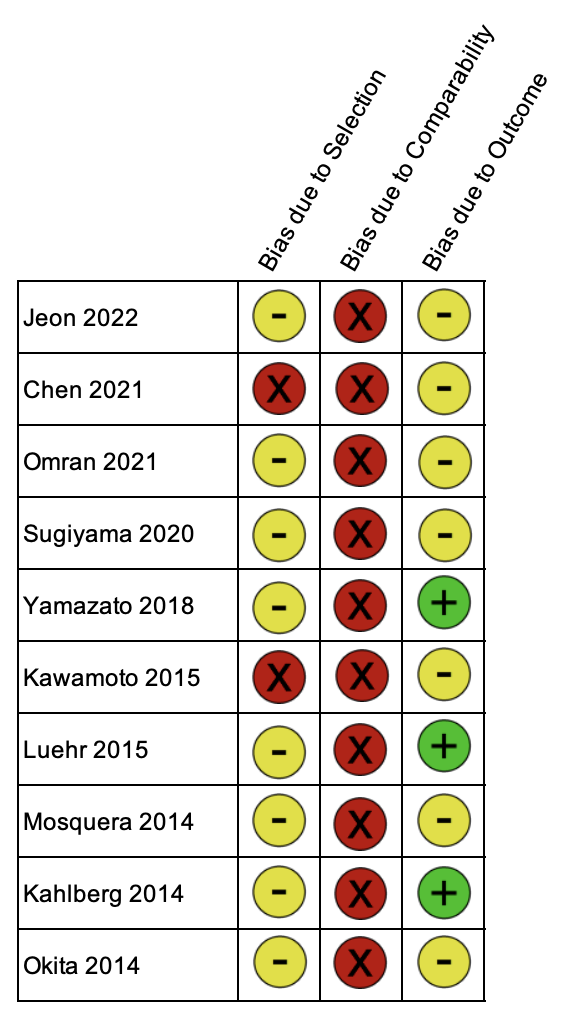


**Supplementary Figure 2 – Risk of bias analysis, conducted by Newcastle - Ottawa Quality Assessment Scale for Case Control Studies.**

Bias due to Selection – Domain scoring 0-1 (high) 2 (some concerns) 3-4 (low)

Bias due to Comparability – Domain scoring 0 (high) 1 (some concerns) 2 (low)

Bias due to Selection – Domain scoring 0 (high) 1 (some concerns) 2-3 (low)
